# Functional Connectivity Features of Resting-State Functional Magnetic Resonance Imaging May Distinguish Migraine From Tension-Type Headache

**DOI:** 10.3389/fnins.2022.851111

**Published:** 2022-04-26

**Authors:** Yajuan Wang, Yingshuang Wang, Lihong Bu, Shaoyang Wang, Xinhui Xie, Fuchun Lin, Zheman Xiao

**Affiliations:** ^1^Department of Neurology, Renmin Hospital of Wuhan University, Wuhan, China; ^2^Positron Emission Tomography-Computer Tomography (PET-CT)/Magnetic Resonance Imaging (MRI) Center, Renmin Hospital of Wuhan University, Wuhan, China; ^3^Department of Emergency, People’s Hospital of Rizhao, Rizhao, China; ^4^Department of Psychiatry, Renmin Hospital of Wuhan University, Wuhan, China; ^5^State Key Laboratory of Magnetic Resonance and Atomic and Molecular Physics, National Center for Magnetic Resonance in Wuhan, Wuhan Institute of Physics and Mathematics, Innovation Academy for Precision Measurement Science and Technology, Chinese Academy of Sciences, Wuhan, China

**Keywords:** migraine, tension-type headache, cognition, emotion, functional connectivity, resting state functional magnetic resonance image

## Abstract

**Background:**

Migraineurs often exhibited abnormalities in cognition, emotion, and resting-state functional connectivity (rsFC), whereas patients with tension-type headache (TTH) rarely exhibited these abnormalities. The aim of this study is to explore whether rsFC alterations in brain regions related to cognition and emotion could be used to distinguish patients with migraine from patients with TTH.

**Methods:**

In this study, Montreal Cognitive Assessment (MoCA), Self-Rating Anxiety Scale (SAS), Self-Rating Depression Scale (SDS), and rsFC analyses were used to assess the cognition, anxiety, and depression of 24 healthy controls (HCs), 24 migraineurs, and 24 patients with TTH. Due to their important roles in neuropsychological functions, the bilateral amygdala and hippocampus were chosen as seed regions for rsFC analyses. We further assessed the accuracy of the potential rsFC alterations for distinguishing migraineurs from non-migraineurs (including HCs and patients with TTH) by the receiver operating characteristic (ROC) analysis. Associations between headache characteristics and rsFC features were calculated using a multi-linear regression model. This clinical trial protocol has been registered in the Chinese Clinical Trial Registry (registry number: ChiCTR1900024307, Registered: 5 July 2019-Retrospectively registered, http://www.chictr.org.cn/showproj.aspx?proj=40817).

**Results:**

Migraineurs showed lower MoCA scores (*p* = 0.010) and higher SAS scores (*p* = 0.017) than HCs. Migraineurs also showed decreased rsFC in the bilateral calcarine/cuneus, lingual gyrus (seed: left amygdala), and bilateral calcarine/cuneus (seed: left hippocampus) in comparison to HCs and patients with TTH. These rsFC features demonstrated significant distinguishing capabilities and got a sensitivity of 82.6% and specificity of 81.8% with an area under the curve (AUC) of 0.868. rsFC alterations showed a significant correlation with headache frequency in migraineurs (*p* = 0.001, *Pc* = 0.020).

**Conclusion:**

The rsFC of amygdala and hippocampus with occipital lobe can be used to distinguish patients with migraine from patients with TTH.

**Clinical Trial Registration:**

[http://www.chictr.org.cn/showproj.aspx?proj=40817], identifier [ChiCTR1900024307].

## Introduction

Migraine is the second most common primary headache disorder after tension-type headache (TTH) (Stovner et al., 2007), and the second leading cause of disability worldwide (Feigin et al., 2019). Migraine and TTH exhibit many similarities in clinical practice (Vargas, 2008). Approximately 37% of patients who were initially diagnosed with TTH developed migraine-like attacks in the late stage (Lipton et al., 2002). Because functional impairments, such as neuropsychological dysfunction and neuroimaging abnormalities, caused by migraine are more serious than those caused by TTH, it is urgent to distinguish patients with migraine from patients with TTH.

Migraineurs often exhibited abnormalities in cognition, psychological function, and resting-state functional connectivity (rsFC), whereas patients with TTH rarely exhibited these functional impairments (Vuralli et al., 2018; Skorobogatykh et al., 2019). The interaction between neuropsychological performance and neuroimaging features may be one of the potential characteristics to distinguish patients with migraine from patients with TTH. The amygdala and hippocampus are the key brain regions related to cognition and emotion (Montagne et al., 2019; de Carvalho et al., 2021; Dogra et al., 2021; Duan et al., 2021; Mateus-Pinheiro et al., 2021; Nguyen et al., 2021). The alterations in rsFC of the two brain regions have been reported in migraineurs when compared to healthy controls (HCs) (Hadjikhani et al., 2013; Zhu et al., 2021). However, there is a lack of studies to directly compare functional brain connectivity between migraine and TTH. As a result, it remains unknown whether these abovementioned rsFC alterations are specific to migraine or just a general marker of recurrent episodes of headache. To clarify this issue, we have performed the current study.

In this study, we conducted neuropsychological tests and seed-based rsFC analyses on age-, sex- and educational years-matched HCs, migraineurs, and patients with TTH. The bilateral amygdala and hippocampus were chosen as seed regions because of their important roles in cognition and emotion. We further assessed the accuracy of the potential rsFC alterations for distinguishing migraineurs from non-migraineurs (including HCs and patients with TTH) by the receiver operating characteristic (ROC) analysis. Moreover, we examined their associations with headache characteristics in migraine and TTH groups. The aim of this study is to explore whether the rsFC alterations with brain regions (amygdala and hippocampus) related to cognition and emotion can be used to distinguish patients with migraine from patients with TTH.

## Materials and Methods

### Participants

All participants were recruited from the Renmin Hospital of Wuhan University from July 2018 to December 2019. A total of 24 HCs, 24 patients with migraine, and 24 patients with TTH were included in this study. The age, gender ratio, and years of education were matched across the three groups. Patients with migraine and patients with TTH were diagnosed by two neurologists according to the International Classification of Headache Disorders, 3th Edition (ICHD-3) (Olesen et al., 2018). Patients were included if they: (1) were between 18 and 60 years old and had more than 6 years of education, (2) experienced headache at least once a month in the last 3 months, and (3) had a history of migraine or TTH for at least 6 months. Patients were excluded if they: (1) had contraindications for magnetic resonance imaging (MRI), (2) had any other diseases in addition to migraine or TTH, (3) had a history of alcohol or drug abuse, and (4) were pregnant or lactating. HCs had no known diseases. The exclusion criteria for HCs were the same as those for patients. To minimize the effects of an impending headache or a prior headache, all patients were headache-free for at least 72 h at the time of the MRI scan. Our clinical trial protocol has been registered in the Chinese Clinical Trial Registry(registry number: ChiCTR1900024307).^1^

### Assessment of Cognition, Anxiety, and Depression

The cognitive function of participants was assessed using the Montreal Cognitive Assessment (MoCA), which evaluated six cognitive domains with a total score of 30, including visuospatial/executive functions, naming, attention, language, abstraction, delay recall, and orientation. A MoCA score ≥26 indicates normal cognition. Participants’ anxiety and depression states were assessed using the Self-Rating Anxiety Scale (SAS) and Self-Rating Depression Scale (SDS), respectively. The higher the scores of SAS and SDS, the more serious the symptoms of anxiety and depression.

### Magnetic Resonance Imaging Acquisition

Resting-state functional MRI (fMRI) scans were acquired on a General Electric (Signa HDxt) 3.0T scanner, which had a standard 8-channel head coil and used echo-planar imaging with the following parameters: repetition time = 2,000 ms; echo time = 30 ms; flip angle = 90^°^; acquisition matrix = 64 × 64; field of view = 220 mm × 220 mm; and slice thickness = 4 mm with a 0.6-mm gap. Each volume consisted of 31 axial slices, and each run contained 240 volumes. During fMRI scanning, all subjects were instructed to close their eyes and rest, and not to think about anything or fall asleep.

### Image Pre-processing

Image pre-processing was performed using the DPARSF software.^2^ The first 10 volumes were discarded to avoid signal instability. Slice-timing and head-motion correction were conducted on the remaining 230 volumes. We used the Friston 24 parameter model to eliminate the effects of head motion (Friston et al., 1996). Any participants with head motion greater than 2.5 mm or 2.5° in any direction were excluded from this study. As a result, one HC, one migraineur, and two patients with TTH were discarded, and a total of 23 HCs, 23 migraineurs, and 22 patients with TTH were ultimately included for further analyses. We compared framewise displacement (FD) among the HC, migraine, and TTH groups to avoid the effect of microscopic head motions. The mean FD scores did not differ among the three groups (*p* > 0.05) and were used as a covariate for intergroup comparisons. Then, the realigned images were normalized to the Montreal Neurological Institute (MNI) space, resampled to a 3-mm isotropic voxel, and smoothened with a 4-mm full width at half maximum isotropic Gaussian kernel. After these steps, the processed data were detrended, and the white matter and cerebrospinal fluid signals were removed by a regression analysis. Finally, temporal bandpass filtering (0.01–0.1 Hz) was applied to reduce the effects of low-frequency drift and high-frequency noise.

### Seed-Based Resting-State Functional Connectivity Analyses

Previous studies have shown that migraineurs exhibited alterations in the rsFC of amygdala and hippocampus (Hadjikhani et al., 2013; Zhu et al., 2021), and the two brain regions were associated with cognition and emotion (Montagne et al., 2019; de Carvalho et al., 2021; Dogra et al., 2021; Duan et al., 2021; Mateus-Pinheiro et al., 2021; Nguyen et al., 2021). Considering the differences in neuropsychological performance between migraine and TTH (Waldie et al., 2002; Gil-Gouveia et al., 2015, 2016; Huang et al., 2017; Puledda et al., 2017; Karsan and Goadsby, 2018; Vuralli et al., 2018), we chose the bilateral amygdala and hippocampus as seed regions for rsFC analyses, to explore the potential rsFC features that may distinguish the two types of headache. The average time series were calculated for each seed in each subject. Then, the Pearson correlation coefficient was calculated between the average time course of the seed and that of each voxel of the whole brain. A Fisher’s *z*-transformation was applied to improve the normality of the correlation coefficient (Wang et al., 2006). Finally, the rsFC maps of each seed were obtained for each subject.

### Statistical Analysis

We used the R software (R version 3.6.1) for statistical analysis. Potential differences in demographic and psychometric among the HC, migraine, and TTH groups were evaluated using one-way analysis of variance (ANOVA) and *post-hoc* analysis for continuous variables, and chi-squared tests for categorical variables. Potential differences in headache characteristics between migraine and TTH groups were evaluated using Welch’s two-sample *t*-test. The value of *p* < 0.05 was considered as statistically significant.

A whole-brain voxel-wise analysis of intergroup differences in the rsFC for each seed was performed using one-way analysis of covariance (ANCOVA), with age, gender, years of education, and mean FD as covariates. Statistical differences were set at a threshold of false discovery rate (FDR) corrected *p* < 0.05 at the voxel level. *Post-hoc* multiple comparisons were performed on the clusters that showed significant differences in one-way ANCOVA using Tukey’s test, to test pair-wise differences between the groups (HC vs. migraine, HC vs. TTH, and migraine vs. TTH). The accuracy of the potential rsFC alterations for distinguishing migraineurs from non-migraineurs (including HCs and patients with TTH) was assessed using the ROC analysis. The optimal cut-off for classifying migraineurs vs. non-migraineurs based on these rsFC alterations was calculated using Youden’s index (*J* = sensitivity + specificity–1). The optimal cut-off was determined as the point with the maximum index value.

Additionally, the multiple linear regression model was performed to examine the associations between headache characteristics and the altered rsFC. In the model, the averaged rsFC strengths in the significant regions were used as dependent variables, and headache characteristics (disease duration, headache frequency, single-attack duration, and headache intensity) were used as independent variables, with age, gender, and years of education as covariates. A threshold of α = 0.05 was applied to consider regression weights significant, and the Bonferroni correction was used for multiple comparisons.

## Results

### Basic Characteristics and Intergroup Comparisons

**FIGURE 1:**
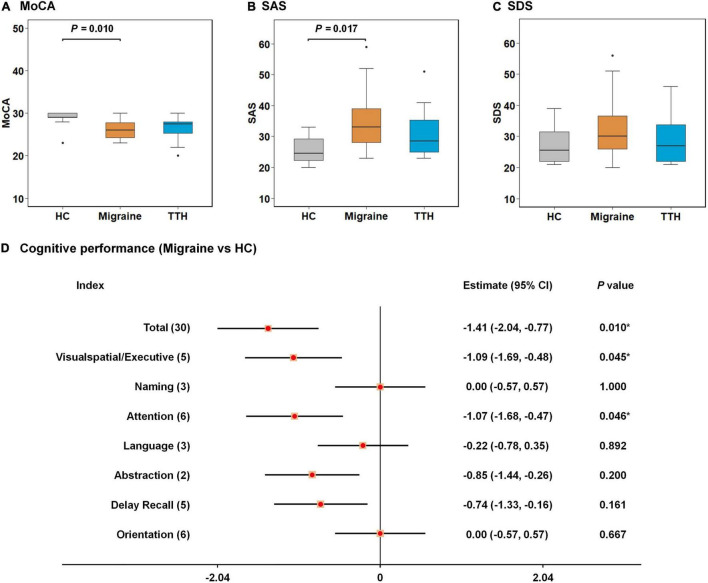
Intergroup differences in neuropsychological tests among healthy control (HC), migraine, and TTH groups. Migraineurs showed lower scores on MoCA **(A)** and higher scores on SAS **(B)** than HCs; no significant differences in SDS were observed among the three groups **(C)**; the affected cognitive domains in migraineurs were visuospatial/executive functions and attention in comparison with HCs **(D)**. The intergroup differences were tested using one-way ANOVA and *post-hoc* analysis. Significant *p*-values for each pair of intergroup comparisons were depicted at the top of each figure **(A–C)**; differences in cognitive performances between migraineurs and HCs were assessed by Welch’s two-sample *t*-test, and were demonstrated by 95% CI and *p*-values. *p-value < 0.05 **(D)**. TTH, tension-type headache; MoCA, Montreal Cognitive Assessment; SAS, self-rating anxiety Scale; SDS, self-rating depression scale; ANOVA, analysis of variance; CI, confidence interval.

The demographic, psychometric, and headache characteristics of our study population (*n* = 72, including 24 HCs, 24 migraines, and 24 TTH) are summarized in Table 1. Notably, demographic and clinical characteristics were analyzed for all study subjects (*n* = 72) although only 68 subjects were ultimately used for the rsFC analysis due to the exclusion of one HC, one migraine patient, and two patients with TTH after head-motion control. The study population had a female proportion of 68%, an age range from 18 to 54 years old at baseline (mean ± SD: 33.4 ± 8.7) and education of 14.2 ± 2.6 years. The difference in age, gender ratio, and educational years among the three groups did not reach statistical significance (*p* > 0.05; Table 1). Cognitive performances (i.e., MoCA) significantly differed among the three groups (*p* = 0.010; Table 1) with lower scores on MoCA in migraine than HC (*p* = 0.010; Figure 1A), where lower scores represent a worse cognitive function. The affected cognitive domains in migraineurs were visuospatial/executive functions (*p* = 0.045; Figure 1D) and attention (*p* = 0.046; Figure 1D) in comparison with HCs. Participants’ anxiety states (i.e., SAS) significantly differed among the three groups (*p* = 0.022; Table 1) with higher scores on SAS in migraine than HC (*p* = 0.017; Figure 1B), where higher scores represent more severe anxiety symptom. The difference in participants’ depression states (i.e., SDS) among the three groups did not reach statistical significance (*p* > 0.05; Table 1, Figure 1C). There were no significant differences in MoCA, SAS, and SDS between TTH and HCs. Headache characteristics of migraine and TTH were assessed in terms of disease duration, headache frequency, single-attack duration, and headache intensity. Migraineurs exhibited less frequent episodes (*p* = 0.016; Table 1), a longer single-attack duration (*p* = 0.043; Table 1), and a higher headache intensity (*p* < 0.001; Table 1) in comparison with TTH. There were no significant differences in the disease duration between migraine and TTH groups.

**TABLE 1:** Demographic, psychometric, and headache characteristics of all participants.

	HC (*n* = 24)	Migraine (*n* = 24)	TTH (*n* = 24)	*F/*χ*^2^/t*	*P-*value	η*^2^/Cohen’s φ /Cohen’s d*
Age (years)	33.29 ± 9.34	30.75 ± 6.89	36.21 ± 9.23	2.442*^a^*	0.095*^a^*	0.066*^a^*
Female (%)	14 (58%)	18 (75%)	17 (71%)	1.661*^b^*	0.436*^b^*	0.263*^b^*
Educational level (years)	15.08 ± 2.19	14.29 ± 3.03	13.33 ± 2.37	2.825*^a^*	0.066*^a^*	0.076*^a^*
MoCA	28.73 ± 2.00	25.94 ± 1.95	26.33 ± 3.06	5.180*^a^*	0.010^a^*	0.131*^a^*
SAS	25.60 ± 4.48	35.16 ± 9.81	31.50 ± 8.49	4.210*^a^*	0.022^a^*	0.109*^a^*
SDS	27.60 ± 6.95	32.53 ± 9.49	29.08 ± 8.59	1.225*^a^*	0.305*^a^*	0.034*^a^*
Disease duration (years)	NA	6.80 ± 4.28	5.00 ± 5.15	1.288*^c^*	0.205*^c^*	0.380*^c^*
Headache frequency (*n*/month)	NA	2.25 ± 1.85	7.75 ± 10.20	−2.597*^c^*	0.016^c^*	−0.750*^c^*
Single-attack duration (hours)	NA	17.39 ± 17.52	8.52 ± 9.33	2.104*^c^*	0.043^c^*	0.632*^c^*
Headache intensity (0–10)	NA	7.23 ± 1.53	4.96 ± 1.32	5.154*^c^*	<0.001^c^*	1.589*^c^*

*Categorical variables are reported as numbers and percentages; continuous variables are reported as means ± standard deviations (SDs). Demographic and clinical characteristics were analyzed for all study subjects (n = 72), although only 68 subjects were ultimately subjected to resting-state functional connectivity (rsFC) analysis due to the exclusion of one HC, one migraine patient, and two TTH patients after head-motion control.*

*^a^F-values,*

*^a^p-values, and*

*^a^η^2^ for the age, educational level, and neuropsychological scores in the three groups were obtained using one-way ANOVA.*

*^b^χ^2^-values,*

*^b^p-values, and*

*^b^cohen’s φ for the gender distribution in the three groups were obtained using chi-squared analysis.*

*^c^T-values,*

*^c^p-values, and*

^c^cohen’s d for the headache characteristics in migraine and TTH groups were obtained using Welch’s two-sample t-test. *p-value < 0.05. HC, healthy control; TTH, tension-type headache; MoCA, Montreal Cognitive Assessment; SAS, self-rating anxiety scale; SDS, self-rating depression scale; ANOVA, analysis of variance.

### Seed-Based Resting-State Functional Connectivity

A significant result from one-way ANCOVA for the seed-based rsFC among HC, migraine, and TTH groups is shown in Table 2. Using the left amygdala as a seed, there were significant differences in rsFC with the bilateral calcarine/cuneus and bilateral lingual gyrus. For the left hippocampus, there were significant differences in rsFC with the bilateral calcarine/cuneus. No significant differences in rsFC were observed using the right amygdala or the right hippocampus as seeds.

**TABLE 2:** Brain regions showing significant differences in rsFC among the HC, migraine and TTH groups.

Seed	Regions of differences	Peak coordinates (MNI)			Cluster size (voxels)	Peak *F*	η^2^
		X	Y	Z			
Amy_L	Bilateral calcarine/cuneus	−3	−72	18	568	18.87	0.354
	Left lingual gyrus	−21	−66	−18	75	11.74	0.254
	Right lingual gyrus	15	−69	−18	37	11.03	0.242
Hip_L	Left calcarine/cuneus	−15	−60	15	148	17.02	0.330
	Right calcarine/cuneus	12	−63	15	39	11.67	0.253

*The intergroup differences among the three groups were tested using one-way ANCOVA with age, gender, educational years, and mean FD values as covariates. A threshold of p < 0.05 (FDR corrected) at voxel level was considered statistically different. Amy_L, left amygdala; Hip_L, left hippocampus; MNI, Montreal Neurological Institute; HC, healthy control; TTH, tension-type headache; ANCOVA, analysis of covariance; FD, framewise displacement; FDR, false discovery rate.*

**FIGURE 2:**
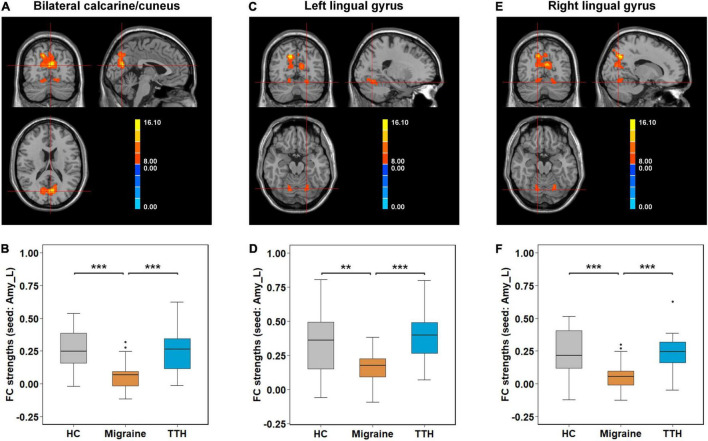
Intergroup differences in rsFC strengths (seed: left amygdala). Migraineurs showed decreased rsFC of left amygdala with bilateral calcarine/cuneus (–3,–72, 18) **(A,B)**, left lingual gyrus (–21,–66,–18) **(C,D)**, and right lingual gyrus (15,–69,–18) **(E,F)** in comparison with HCs and patients with TTH. The pair-wise differences in the significant clusters observed in one-way ANCOVA (Table 2) were tested using *post-hoc t*-tests. *p-value < 0.05; **p-value < 0.01; ***p-value < 0.001. rsFC, resting-state functional connectivity; Amy_L, left amygdala; HC, healthy control; TTH, tension-type headache; ANCOVA, analysis of covariance.

**FIGURE 3:**
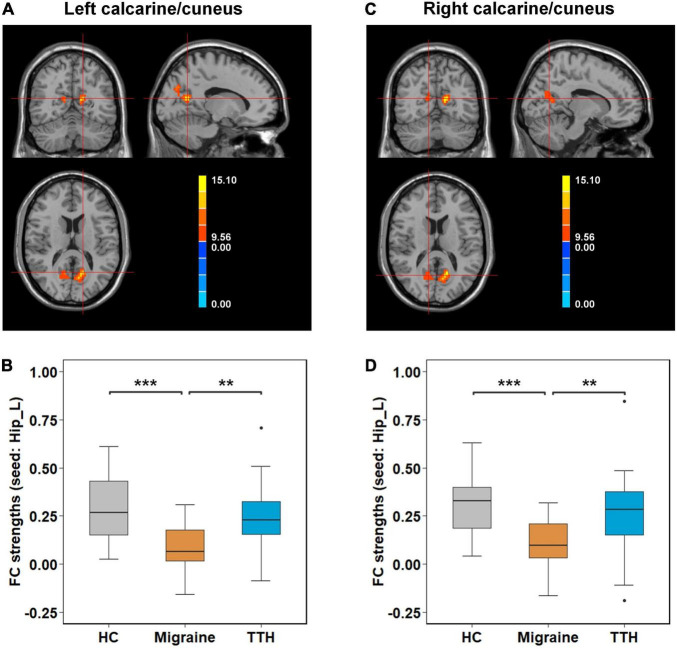
Intergroup differences in rsFC strengths (seed: left hippocampus). Migraineurs showed decreased rsFC of left hippocampus with left calcarine/cuneus (–15,–60, 15) **(A,B)** and right calcarine/cuneus (12,–63, 15) **(C,D)** in comparison with HCs and patients with TTH. The pair-wise differences in the significant clusters observed in one-way ANCOVA (Table 2) were tested using *post-hoc t*-tests. *p-value < 0.05; **p-value < 0.01; ***p-value < 0.001. rsFC, resting-state functional connectivity; Hip_L, left hippocampus; HCs, healthy controls; TTH, tension-type headache; ANCOVA, analysis of covariance.

*Post-hoc t*-tests were then performed on the clusters that showed significant differences in one-way ANCOVA, to test pair-wise differences between the groups (HC vs. migraine, HC vs. TTH, and migraine vs. TTH). Using the left amygdala as a seed, migraineurs showed decreased rsFC with the bilateral calcarine/cuneus (compared to HC: *p* < 0.001; compared to TTH: *p* < 0.001; Figures 2A,B), left lingual gyrus (compared to HC: *p* = 0.003; compared to TTH: *p* < 0.001; Figures 2C,D) and right lingual gyrus (compared to HC: *p* < 0.001; compared to TTH: *p* < 0.001; Figures 2E,F). Using the left hippocampus as a seed, patients with migraine showed decreased rsFC with the left calcarine/cuneus (compared to HC: *p* < 0.001; compared to TTH: *p* = 0.003; Figures 3A,B) and right calcarine/cuneus (compared to HC: *p* < 0.001; compared to TTH: *p* = 0.003; Figures 3C,D). There were no significant differences in rsFC between HCs and patients with TTH.

### Classification of Migraineurs and Non-migraineurs Based on the Altered Resting-State Functional Connectivity

**FIGURE 4:**
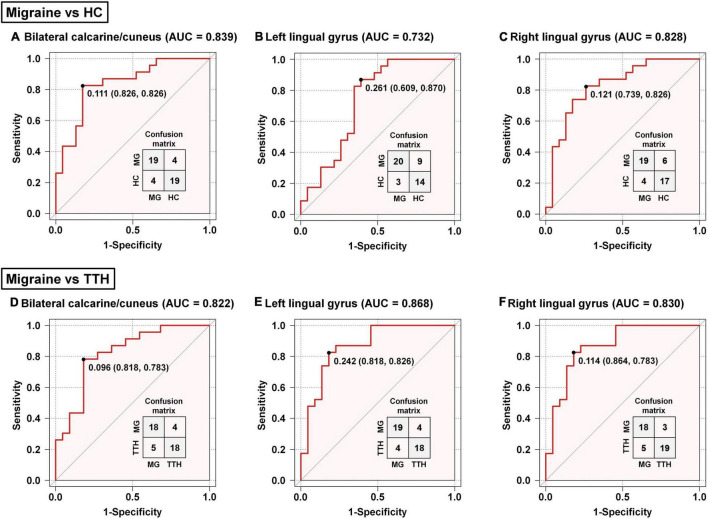
Classification of migraineurs and non-migraineurs based on the rsFC alterations (seed: left amygdala). Classification of migraineurs and HCs **(A–C)**; classification of migraineurs and patients with TTH **(D–F)**. The point on each ROC curve represents the optimal cut-off (specificity and sensitivity). MG, migraine; HC, healthy controls; TTH, tension-type headache; ROC, receiver operating characteristic; AUC, area under the curve.

The accuracy of these rsFC alterations in discriminating migraineurs from non-migraineurs including (HCs and patients with TTH) was assessed by the ROC analysis. Based on the rsFC between the left amygdala and bilateral calcarine/cuneus, the area under the curve (AUC), optimal cut-off, sensitivity, and specificity of discriminating migraineurs from HCs (Figure 4A) and of discriminating migraineurs from patients with TTH (Figure 4D) were 0.839, 0.111, 82.6%, and 82.6% and 0.822, 0.096, 78.3%, and 81.8%, respectively. Based on the rsFC between the left amygdala and left lingual gyrus, the AUC, optimal cut-off, sensitivity, and specificity of discriminating migraineurs from HCs (Figure 4B) and for discriminating migraineurs from patients with TTH (Figure 4E) were 0.732, 0.261, 87%, and 60.9% and 0.868, 0.242, 82.6%, and 81.8%, respectively. Based on the rsFC between the left amygdala and right lingual gyrus, the AUC, optimal cut-off, sensitivity, and specificity of discriminating migraineurs from HCs (Figure 4C) and of discriminating migraineurs from patients with TTH (Figure 4F) were 0.828, 0.121, 82.6%, and 73.9% and 0.830, 0.114, 78.3%, and 86.4%, respectively.

**FIGURE 5:**
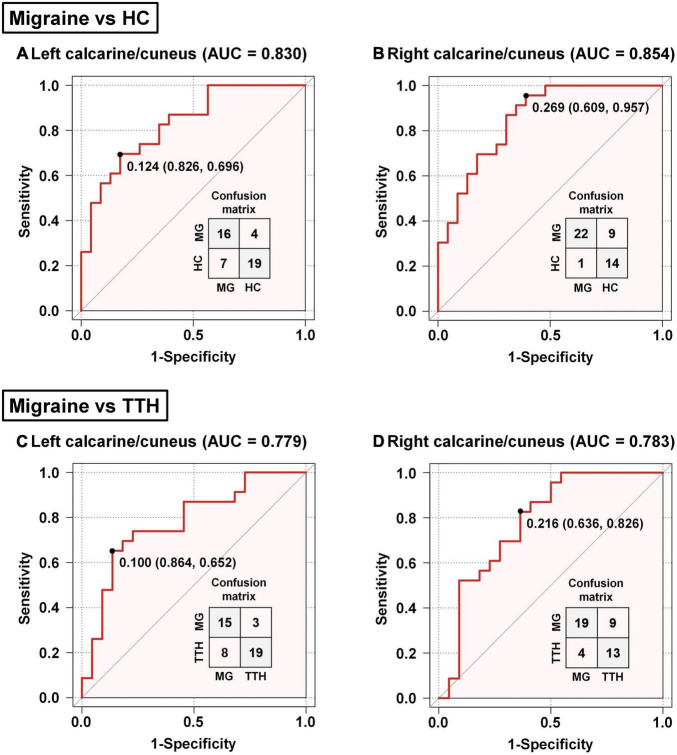
Classification of migraineurs and non-migraineurs based on the rsFC alterations (seed: left hippocampus). Classification of migraineurs and HCs **(A,B)**; classification of migraineurs and patients with TTH **(C,D)**. The point on each ROC curve represents the optimal cut-off (specificity and sensitivity). MG, migraine; HC, healthy controls; TTH, tension-type headache; ROC, receiver operating characteristic; AUC, area under the curve.

Based on the rsFC between the left hippocampus and left calcarine/cuneus, the AUC, optimal cut-off, sensitivity, and specificity were 0.830, 0.124, 69.6%, and 82.6% for discriminating migraineurs from HCs (Figure 5A), and 0.779, 0.100, 65.2%, and 86.4% for discriminating migraineurs from patients with TTH (Figure 5C). Based on the rsFC between the left hippocampus and right calcarine/cuneus, the AUC, optimal cut-off, sensitivity, and specificity were 0.854, 0.269, 95.7%, and 60.9% for discriminating migraineurs from HCs (Figure 5B), and 0.783, 0.216, 82.6%, and 63.6% for discriminating migraineurs from patients with TTH (Figure 5D).

### Associations Between Headache Characteristics and the Altered Resting-State Functional Connectivity

**FIGURE 6:**
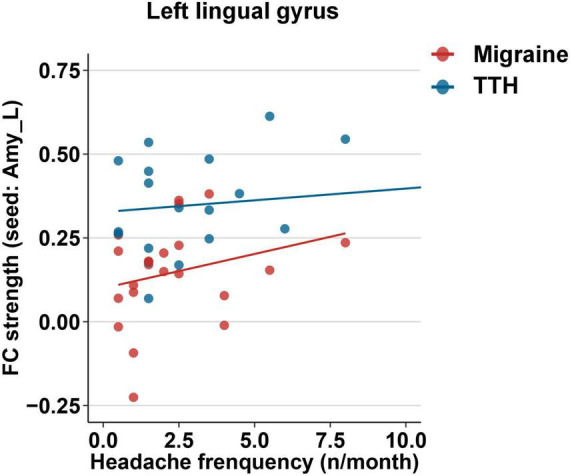
Associations between the rsFC strength of the left amygdala with the left lingual gyrus and headache frequency in migraine (red) and TTH (blue) groups. Only the association in migraine group (red) reached statistical significance after Bonferroni correction. The associations of headache characteristics with rsFC strengths in the significant regions were tested using multiple linear regression model with age, gender, and educational years as covariates. Specific β coefficients and *p*-values can be found in Additional File 1. TTH, tension-type headache.

Next, we examined associations between headache characteristics (including disease duration, headache frequency, single-attack duration, and headache intensity) and the altered rsFC in migraine and TTH groups. In the migraine group, the rsFC strength of the left amygdala with the bilateral calcarine/cuneus was associated with headache frequency (*p* = 0.029); the rsFC strength of the left amygdala with the left lingual gyrus was associated with disease duration (*p* = 0.030), headache frequency (*p* = 0.001, *Pc* = 0.020, Figure 6), single-attack duration (*p* = 0.012), and headache intensity (*p* = 0.011); the rsFC strength of the left amygdala with the right lingual gyrus was associated with headache frequency (*p* = 0.049). However, in the migraine group, only the association of headache frequency with rsFC within the left amygdala and left lingual gyrus survived the Bonferroni correction (Figure 6). In the TTH group, there were no significant associations between headache characteristics and rsFC strengths. Using the left hippocampus as a seed, there were no significant associations between headache characteristics and rsFC strengths in the migraine or TTH group. Specific β coefficients and *p-*values can be found in Additional File 1.

## Discussion

In this study, we found that compared with HCs and patients with TTH, migraineurs exhibited impaired cognition and increased anxiety, which showed lower MoCA scores and higher SAS scores. The rsFC features of amygdala and hippocampus with the occipital lobe, such as the bilateral calcarine/cuneus and lingual gyrus, could significantly distinguish migraineurs from non-migraineurs (including HCs and patients with TTH). Moreover, the rsFC strength of the left amygdala with the left lingual gyrus was associated with headache frequency in migraineurs. These findings suggested that the rsFC features might be applied into clinical practice in the future to distinguish migraineurs from patients with TTH.

### Impaired Cognition and Increased Anxiety in Migraineurs

Migraineurs frequently experienced cognitive and psychological dysfunction, such as difficulty in concentration, anxiety, and unhappiness (Gil-Gouveia et al., 2015; Huang et al., 2017; Puledda et al., 2017; Karsan and Goadsby, 2018; Vuralli et al., 2018). Impaired cognitive and psychological functions may distinguish migraineurs from patients with other types of headaches (Vuralli et al., 2018). Although TTH is the most common primary headache disorder, the neuropsychological performance in patients with TTH has not been thoroughly investigated as patients with migraine. Two prospective comparative studies showed that the cognitive and psychological functions of patients with TTH were similar to those of HCs, but different from those from migraineurs (Waldie et al., 2002; Gil-Gouveia et al., 2016). In this study, we found that migraineurs exhibited impaired cognition, which was characterized by the impairment of visuospatial/executive functions and attention, as well as increased anxiety, which were generally consistent with previous studies (Gil-Gouveia et al., 2015; Huang et al., 2017; Puledda et al., 2017; Karsan and Goadsby, 2018; Vuralli et al., 2018). Additionally, the neuropsychological scores (i.e., MoCA, SAS, and SDS) of patients with TTH were intermediate between those of migraineurs and HCs. These findings confirmed that impaired cognition and increased anxiety were more severe in patients with migraine than in patients with TTH. Therefore, we suppose that the brain functional changes with brain regions related to cognition and emotion may distinguish patients with migraine from patients with TTH.

### Resting-State Functional Connectivity Alterations in Migraineurs and Their Discriminative Ability

As a hypothesis-driven fMRI study, the bilateral amygdala and hippocampus, which are known to be associated with cognition and emotion in well-replicated studies (Montagne et al., 2019; de Carvalho et al., 2021; Dogra et al., 2021; Duan et al., 2021; Mateus-Pinheiro et al., 2021; Nguyen et al., 2021), were selected as seed regions for subsequent rsFC analyses. Our results indicated that migraineurs showed decreased rsFC mainly in the occipital lobe, such as the bilateral calcarine/cuneus and lingual gyrus, using the left amygdala and left hippocampus as seeds. Similarly, the rsFC alterations in the occipital lobe have been identified as the most specific imaging markers to distinguish patients with migraine from HCs or patients with other chronic pain disorders, such as chronic low back pain and fibromyalgia, using fMRI-based machine learning approach (Tu et al., 2020) and network mapping technique (Burke et al., 2020). However, previous studies did not include other types of headache. As a result, they did not reveal whether these alterations in rsFC are specific to migraine or a general marker of recurrent episodes of headache. In this study, we found that the altered rsFC of the amygdala and hippocampus with the occipital lobe can be used to distinguish migraineurs from not only the HCs but also patients with TTH. When distinguishing migraineurs from HCs, the rsFC between the left hippocampus and right calcarine/cuneus achieved the highest AUC. When distinguishing migraineurs from patients with TTH, the rsFC between the left amygdala and left lingual gyrus achieved the highest AUC. Notably, previously observed rsFC abnormalities in migraineurs did not exist in patients with TTH, suggesting that these abnormalities may be unique to migraine, not just a general sign of recurrent headache.

When the right amygdala and right hippocampus were selected as seeds, we did not observe any difference in rsFC among the three groups. This may be related to the inherent left lateralization of the brain of migraine patients, which is consistent with previous neuroimaging studies (Maniyar et al., 2014; Gaist et al., 2018; Burke et al., 2020). A positron emission tomography (PET) study investigated photic hypersensitivity of migraineurs and reported peak hypermetabolism mainly occurred in the left extrastriate cortex (Maniyar et al., 2014), and a cortical thickness study identified increased left visual cortex thickness of migraine patients (Gaist et al., 2018). Moreover, a recent voxel-based morphometry (VBM) meta-analysis reported that migraine atrophy coordinates were mainly connected to a cluster in the left visual cortex (Burke et al., 2020). Future studies are needed to explore the significance of left lateralization.

### A Correlation Analysis Between Headache Characteristics and Resting-State Functional Connectivity Alterations

Further, a correlation analysis showed that the rsFC strength of the left amygdala with the left lingual gyrus was associated with headache frequency in migraineurs. This result supported previous findings that the migraine attack frequency could be predicted by fMRI-based machine learning approaches (Mu et al., 2020; Tu et al., 2020). In clinical practice, the migraine attack frequency is often assessed by self-report, resulting in the measurement of headache frequency to become inaccurate and unreliable (Berger et al., 2018; Haywood et al., 2018). Because attack frequency is a risk factor for migraine progression, objective measurements are needed to accurately estimate migraine progression. Our results and previous finding (Mu et al., 2020; Tu et al., 2020) suggested that neuroimaging markers might be used to predict factors for the estimation of migraine progression.

### Limitations

There are several limitations to our study. Firstly, we did not include other primary headache disorders, such as cluster headache or other trigeminal autonomic cephalalgias. We included patients with TTH in our study because TTH is the most common primary headache disorder and the most common misdiagnosed migraine. We also included patients with other primary headache disorders in the next diagnostic trials. Secondly, we used pre-specified seed points as a hypothesis-driven approach to reduce the “researcher degrees of freedom,” but this method might miss some potential rsFC alterations. A combination of hypothesis-driven and data-driven approaches will be more appropriate for future studies with larger sample sizes. Thirdly, we compared changes in rsFC in patients who were not in the current headache episode, but it was not clear whether the changes were persistent during the headache episode. Future studies are needed to explore whether our results were symptom-dependent or trait indicators. Fourthly, our study yielded a relatively high AUC value, implying a good differentiation for migraineurs by using these rsFC changes. Nevertheless, it should be noted that this did not mean that our method can be directly applied to clinical practice. Fifthly, only MoCA, SAS, and SDS were used to assess the cognition, anxiety, and depression of the study population, which would not comprehensively reflect neuropsychological function. More detailed and comprehensive neuropsychological tests are needed in the future. Sixthly, we did not assess whether the participants met the diagnosis of anxiety disorder, potentially allowing the results to be influenced by a comorbid anxiety disorder. Seventhly, the small sample size of our study might reduce the statistical power of the study. A rigorous clinical diagnostic trial with sufficient sample size is needed to further validate our results.

## Conclusion

Compared with HCs and patients with TTH, migraineurs exhibited impaired cognition, increased anxiety, and significant rsFC alterations. The rsFC features of amygdala and hippocampus with occipital lobe could significantly distinguish migraineurs from non-migraineurs (including HCs and patients with TTH) and the rsFC strength of the left amygdala with the left lingual gyrus was associated with headache frequency in migraineurs. These findings offer the possibility of developing objective criteria to distinguish migraine from TTH.

## Data Availability Statement

All data generated or analyzed during this study are included in this article and Supplementary Material.

## Ethics Statement

The studies involving human participants were reviewed and approved by the Clinical Research Ethics Committee of Renmin Hospital of Wuhan University. The patients/participants provided their written informed consent to participate in this study.

## Author Contributions

YaW analyzed the data and drafted the manuscript. YiW and LB collected the data. SW analyzed the data. XX revised the manuscript. FL analyzed the data and revised the manuscript. ZX designed and conceptualized the study. All authors contributed to the article and approved the submitted version.

## Conflict of Interest

The authors declare that the research was conducted in the absence of any commercial or financial relationships that could be construed as a potential conflict of interest.

## Publisher’s Note

All claims expressed in this article are solely those of the authors and do not necessarily represent those of their affiliated organizations, or those of the publisher, the editors and the reviewers. Any product that may be evaluated in this article, or claim that may be made by its manufacturer, is not guaranteed or endorsed by the publisher.

## Footnotes

1
http://www.chictr.org.cn

2
http://rfmri.org/DPARSF

## Abbreviations

ANCOVA: analysis of covariance
ANOVA: analysis of variance
AUC: area under the curve
FD: framewise displacement
FDR: false discovery rate
fMRI: functional MRI
ICHD-3: international classification of headache disorders - 3th Edition
MNI: Montreal Neurological Institute
MoCA: Montreal Cognitive Assessment
MRI: magnetic resonance imaging
ROC: receiver operating characteristic
rsFC: resting-state functional connectivity
SAS: self-rating anxiety scale
SDS: self-rating depression scale
TTH: tension-type headache
VBM: voxel-based morphometry.
