# Supplementary material for: Functional Connectivity Features of Resting-State Functional Magnetic Resonance Imaging May Distinguish Migraine From Tension-Type Headache
Source: Front Neurosci. 2022 Apr 26;16:851111. doi: 10.3389/fnins.2022.851111 (PMC9087040; doi:10.3389/fnins.2022.851111)
Supplement: Supplementary file 1 [file Table_1.DOCX]

Supplementary Material

**Additional file 1 Associations of headache characteristics with the rsFC alterations in migraine and TTH groups**

|  | **Headache Characteristics** | | | | | | | | | | | | | | |
| --- | --- | --- | --- | --- | --- | --- | --- | --- | --- | --- | --- | --- | --- | --- | --- |
| **rsFC** | **Disease duration** | | |  | **Headache Frequency** | | |  | **Single-attack duration** | | |  | **Headache intensity** | | |
|  | *β* | *P* | *Pc* |  | *β* | *P* | *Pc* |  | *β* | *P* | *Pc* |  | *β* | *P* | *Pc* |
| **Migraine** |  |  |  |  |  |  |  |  |  |  |  |  |  |  |  |
| **Seed: Amy_L** |  |  |  |  |  |  |  |  |  |  |  |  |  |  |  |
| Bilateral calcarine/cuneus | 0.005 | 0.404 | - |  | **0.028** | **0.029** | - |  | 0.000 | 0.933 | - |  | -0.025 | 0.145 | - |
| Left lingual gyrus | **0.017** | **0.030** | - |  | **0.013** | **0.001** | **0.020^*^** |  | **0.004** | **0.012** | - |  | **0.051** | **0.011** | - |
| Right lingual gyrus | 0.008 | 0.154 | - |  | **0.022** | **0.049** | - |  | 0.001 | 0.465 | - |  | -0.015 | 0.343 | - |
| **Seed: Hip_L** |  |  |  |  |  |  |  |  |  |  |  |  |  |  |  |
| Left calcarine, cuneus | 0.007 | 0.344 | - |  | 0.020 | 0.195 | - |  | 0.000 | 0.980 | - |  | -0.025 | 0.219 | - |
| Right calcarine, cuneus | -0.001 | 0.918 | - |  | 0.009 | 0.592 | - |  | 0.000 | 0.870 | - |  | -0.010 | 0.637 | - |
| **TTH** |  |  |  |  |  |  |  |  |  |  |  |  |  |  |  |
| **Seed: Amy_L** |  |  |  |  |  |  |  |  |  |  |  |  |  |  |  |
| Bilateral calcarine/cuneus | 0.003 | 0.719 | - |  | 0.004 | 0.288 | - |  | 0.001 | 0.898 | - |  | 0.031 | 0.283 | - |
| Left lingual gyrus | 0.001 | 0.892 | - |  | 0.008 | 0.631 | - |  | 0.008 | 0.095 | - |  | 0.041 | 0.209 | - |
| Right lingual gyrus | 0.001 | 0.862 | - |  | 0.004 | 0.337 | - |  | 0.001 | 0.797 | - |  | 0.038 | 0.157 | - |
| **Seed: Hip_L** |  |  |  |  |  |  |  |  |  |  |  |  |  |  |  |
| Left calcarine, cuneus | 0.001 | 0.910 | - |  | 0.006 | 0.218 | - |  | 0.001 | 0.875 | - |  | 0.018 | 0.602 | - |
| Right calcarine, cuneus | 0.005 | 0.630 | - |  | 0.005 | 0.395 | - |  | 0.001 | 0.869 | - |  | 0.037 | 0.372 | - |

Note. The associations of headache characteristics with rsFC strengths in the significant regions were tested using multiple linear regression model with age, gender and educational years as covariates. Bold text indicates a significant *P* value (*P* < 0.05) when not corrected for Bonferroni test. *^*^Pc* < 0.05 after Bonferroni correction.

Abbreviation. Amy_L = left amygdala; Hip_L = left hippocampus; TTH = tension-type headache.
